# The Relation of Post Diphtheritic Paralysis to Antitoxin

**Published:** 1908-03-07

**Authors:** 


### Medicine.

### THE RELATION OF POST-DIPHTHERITIC PARALYSIS TO ANTITOXIN.

The statement has frequently been made that paralysis is more common in cases of diphtheria treated with antitoxin than it is in cases which get well under other forms of treatment. We doubt whether this is actually the case, but the explanation that has been put forward to account for the supposed fact is that with antitoxin treatment a great many cases, which otherwise would have died, survive the disease, but have been so ill that the penalty for survival is some neuritis. Even those who think that temporary paralysis is more common now than it was before serum treatment was introduced do not attribute the neuritis to the serum itself.

It is probable that there are two quite distinct kinds of post-diphtheritic paralysis, the first including only that of the palate, pharynx, and larynx, the cause here being the local inflammation rather than a true intoxication; the second including paralysis of the limbs, eyes, diaphragm, intercostal muscles, and heart, and attributable to the toxins absorbed into the system from the diphtheritic site. It is difficult to prove that these two kinds are really distinct, but Weill and Deguy have investigated the matter very fully, and they hold this view; moreover, they believe that whereas paralysis of the palate and pharynx is no less frequent now than it used to be, the more generalised paralysis is actually rarer than it was before serum treatment was introduced.

Most statistics do not differentiate between the local and the more distant pareses, but group them together. The following figures are derived from the Metropolitan Asylums Board returns:—

| Year | Number of Cases of Diphtheria | Percentage of Cases shewing any form of paralysis |
|---|---|---|
| Antitoxin not used— | | |
| 1893 | 2,848 | 14.2 |
| Antitoxin beginnin to be used— | | |
| 1894 | 3,666 | 13.1 |
| 1895 | 3,635 | 20.4 |
| 1896 | 4,808 | 20.5 |
| 1897 | 5,637 | 20.5 |
| 1898 | 6,566 | 19.4 |
| 1899 | 7,066 | 20.0 |
| 1900 | 7.195 | 18.5 |
| 1901 | 6,926 | 15.0 |
| 1902 | 6,534 | 17.1 |
| 1903 | 5,072 | 17.1 |
| 1904 | 4,687 | 14.8 |
| 1905 | 4,148 | 12.4 |
| 1906 | 5,218 | 10.9 |

On looking at the table it would appear that the serum treatment first caused a considerable increase in the percentage of paralysis cases, and then in later years led to a considerable diminution. There are two large factors which render the interpretation of the figures particularly difficult, and these are: (1) The greater certainty with which mild cases of diphtheria are diagnosed nowadays by means of cultures from suspicious throats: hence the larger number of cases in the later years; it may be that the inclusion of more mild cases accounts for the diminishing proportion of paralyses; (2) the different dosage and time of administration now employed. Every effort is now made to give the serum upon the first day of the disease, and in large doses, whereas formerly the importance of these two points was not realised; hence the figures for the years 1894-1900 are not strictly comparable with those for the years 1901-1906. It is only possible to draw the general conclusion that, since the administration of the serum has been carried out as it is at present, post-diphtheritic paralysis is at least not more common than it used to be.

Experiments have been carried out upon guinea-pigs and other animals with the view of arriving at exact data in regard to post-diphtheritic paralyses and the influence of serum treatment on them. Ransom has published a considerable amount of work upon this subject, his conclusions being:—

1. That paralysis is to be expected after intoxication with not less than one-fourth of the fatal dose of diphtheria toxin; and that with doses less than one-eighth of the minimum lethal dose no paralysis is noticed.
2. That the larger the dose of toxin the severer is the paralysis, provided the animal survives.
3. That neutralised mixtures of toxin and antitoxin, containing the equivalent of one lethal dose of the former, do not cause paralysis.
4. That antitoxin given fifteen to twenty-four hours after intoxication with doses of the toxin not greater than the lethal dose, exercises a mollifying influence on the subsequent paralysis if the doses of antitoxin are large. Small doses of antitoxin have little effect in diminishing the paralysis.
5. That we may therefore expect liberal doses of antitoxin, given early in the illness in human beings, to have a favourable influence on the subsequent paralysis. Severe cases are likely to be followed by some paralysis in spite of even large doses of antitoxin. The beneficial influence is more likely to manifest itself on such symptoms as failure of the heart than upon the local paralyses.

These conclusions drawn from experimental work upon animals are quite supported by the more recent results found in man. Rolleston has tabulated a large number of Metropolitan Asylums Board cases, according to the day of the disease when the antitoxin was given; the doses of the latter were from 12,000 to 24,000 units. From his table it is quite clear that when serum is given early the tendency to paralysis is very much decreased; that this beneficial effect of the serum affects both local and general paralysis; and that, though the effect is most marked when the serum is given on the first day, it is still evident up to the third, and even the fourth, day, whilst after this the giving of serum does not do much in the way of preventing paralysis. This is precisely parallel to what has been found in connection with the other beneficial effects of anti-diphtheritic serum, and we must unlearn the teaching that serum treatment is apt to predispose to post-diphtheritic paralysis. It is most important to give the serum in large doses upon the earliest possible day in the disease, not only as the best way to save the patient's life, but also to avoid subsequent paralysis.

